# Differential Expression of MicroRNAs in Endarterectomy Specimens Taken from Patients with Asymptomatic and Symptomatic Carotid Plaques

**DOI:** 10.1371/journal.pone.0161632

**Published:** 2016-09-15

**Authors:** Birgit Markus, Karsten Grote, Michael Worsch, Behnoush Parviz, Andreas Boening, Bernhard Schieffer, Mariana S. Parahuleva

**Affiliations:** 1 Internal Medicine/Cardiology and Angiology, University Hospital of Giessen and Marburg, Location Marburg, Germany; 2 Internal Medicine I/Cardiology and Angiology, University Hospital of Giessen and Marburg, Location Giessen, Germany; 3 Department of Cardiovascular Surgery, University Hospital of Giessen and Marburg, Location Giessen, Germany; Brigham and Women's Hospital, Harvard Medical School, UNITED STATES

## Abstract

**Objective:**

Stroke and transient ischemic attacks are considered as clinical manifestations of atherosclerotic disease due to on-going vascular inflammation and finally atherothrombosis of the carotid arteries. MicroRNAs (miRNA/miR) are known to be involved in vascular inflammation and plaque destabilization. The aim of this study was to analyze the expression profile of selected miRNAs in endarterectomy specimen from carotid arteries that were taken from patients with asymptomatic and symptomatic atherosclerotic plaques.

**Methods and Results:**

11 miRNAs were selected and their expression was analyzed using real-time RT-PCR. Therefore, samples were divided into three different groups. On the one hand we investigated the expression patterns from patients in asymptomatic (n = 14) and symptomatic (n = 10) plaques; on the other hand we took samples from normal configurated internal mammary arteries (n = 15). Out of these 11 targets we identified some miRNAs, which were up- or down-regulated in either one of the two groups. Interestingly, the expression of two miRNAs was significantly different between asymptomatic and symptomatic samples, namely miR-21 (P<0.01) and miR-143 (P<0.05).

**Conclusion:**

In the present study, we identified miRNA subtypes which showed different expression in endarterectomy specimen from patients with asymptomatic and symptomatic plaques, suggesting that these miRNAs correlated with advanced vascular inflammation and plaque stability. They may represent new therapeutic targets for vascular proliferative diseases such as atherosclerosis.

## Introduction

The family of non-coding RNAs with a length of ~22 nucleotides, known as microRNAs (miRNA/miR), emerged as key regulators of vascular (patho)-physiological processes while binding to the 3’untranslated region of target mRNAs [1–5]. Increasing evidence supports a role of miRNAs in regulating a variety of ischemic disease-related biological processes, such as myocardial infarction or stroke due to their ability to promote plaque rupture [6–8]. Various miRNAs are known to be expressed in ischemic stroke. Especially circulating miRNAs, which are well-protected from degradation because of their microvesical envelope, have been shown to be associated with the clinical subtypes of stroke and could furthermore be used as biomarkers for ischemic events [9,10]. However, miRNAs have been shown to exhibit diagnostic and therapeutic value in vascular inflammatory diseases and vascular cell damage [11–13]. However, little is known about the miRNA expression profile during atherosclerotic plaque development and destabilization. In this regard, plaque destabilization is a multifactorial process, which is critically driven by inflammatory cells. Mainly the interaction of monocytes and macrophages with cells from the vessel wall [4–6] finally leads to plaque rupture with its clinical manifestation i.e. myocardial infarction or stroke [7]. Therefore, we here evaluated miRNA expression pattern in carotid plaque specimens from patients with symptomatic and asymptomatic plaques. We identified richly expressed miRNAs in carotid plaques using microarray techniques and further analyzed them by real-time RT-PCR. We enrolled patients who had sustained a recent thromboembolic cerebrovascular event (advanced symptomatic carotid plaques) and from patients with asymptomatic carotid plaques. Non-atherosclerotic specimens were obtained from internal mammary arteries and served as a control group. Our observations will identify plaque miRNA candidates, which are correlated with different stages of atherosclerotic vascular disease and may help to develop future therapeutic strategies to modulate plaque progression and stability.

## Subjects and Methods

### Study population

Patients were recruited during the “Factor Seven Activating Protease/FSAP–System in Stroke” study and 24 patients were included in this study as a single center study. On the basis of their clinical characteristics, patients were classified into two diagnostic groups: symptomatic (n = 10) or asymptomatic (n = 14) (Table 1).

**Table 1:** Characteristics of the patients.

Characteristics	Asymptomatic	Symptomatic
Age, years (arithmetic average)	67	69
Female gender, n (%)	4 (28)	3 (30)
**Cardiovascular risk factors, n (%)**		
Smoking	4 (28)	4 (40)
Arterial Hypertension	11 (78)	10 (100)
Dyslipidaemia	9 (64)	6 (60)
Diabetes mellitus	3 (21)	2 (20)
Positive ^c^FHx	4 (28)	1 (10)
**Medical Treatment, n (%)**		
Aspirin/Clopidogr	14 (100)	10 (100)
Statin	14 (100)	6 (60)
^a^ACE-inhibitors/^b^ARB	11 (78)	4 (40)
β-Blocker	9 (64)	3 (30)

^a^ACE: angiotensin-converting enzyme

^b^ARB: AT_1_-rezeptor-blocker

^c^FHx: family history

Presence of symptomatic carotid stenosis was defined as being referred to the stroke unit for symptoms of transient ischemic attack or stroke. At admission, a cerebral computed tomography (CT) scan was performed to exclude intracerebral hemorrhage. Ultrasound examinations were initiated to determine the degree of carotid stenosis and to evaluate the intracerebral vessels. Angiography (magnetic resonance or intra-arterial) was performed to verify the location and extent of a carotid stenosis or occlusion if suspected by ultrasound. Entry criteria were as follows: (1) a transient ischemic attack or stroke ≤6 weeks before carotid endarterectomy; (2) no previous neurologic symptoms before carotid endarterectomy; and (3) internal or common carotid artery (CCA) stenosis >70% defined by velocity criteria on duplex scanning. Exclusion criteria for patients are (1) amaurosis fugax alone or nonfocal, atypical, or distant neurologic symptoms; (2) they had recent (<6 months) carotid endarterectomy; (3) they had recent (<6 months) symptoms of transient ischemic attack and stroke; (4) they were pregnant or breastfeeding; (5) they were unwilling to provide written informed consent to participate; (6) there were contraindications for carotid endarterectomy.

Clinical imaging, age, gender, cardiovascular risk factors including systemic arterial hypertension, diabetes mellitus, smoking, serum characteristics e.g. dyslipidaemia, and family history of coronary artery disease (CAD) as well as medication were assessed and recorded at study entry (Table 1). Patients with high-grade (>70%) symptomatic (n = 10) and asymptomatic (n = 14) carotid stenosis admitted to the Department of Vascular Surgery, University Hospital of Giessen and Marburg, Location Giessen were enrolled into the study and underwent carotid endarterectomy as soon as they were considered stable by the stroke unit. Furthermore, 15 unaffected internal mammary arteries were obtained during coronary bypass surgery and were used as control. All tissue samples were immediately snap frozen in liquid nitrogen and stored at -80°C until use. Hematoxylin-eosin stained sections from each tissue block were examined to establish the morphological characteristics of the plaques, in accordance with the classification of Stary [14]. This study was approved by the Ethics Committee of the University of Giessen and written informed consent was obtained from all subjects.RNA-Isolation

Tissue samples were cut into ~20 μm thick cryostat sections (Leica CM 1900, Leica Microsystems, Wetzlar, Germany) at a temperature of -22°C and stored in 2 ml Eppendorf^®^-tubes. Afterwards, total RNA was extracted from the tissue sections using the Roti^®^-Quick-Kit (Carl Roth GmBH, Karlsruhe, Germany) following the manufacturer's instructions. RNA concentrations were measured using an Eppendorf^®^ BioPhotometer. In addition, we used the SABiosciences RT² qPCR-Grade miRNA Isolation Kit (SABiosciences Corporation, Frederick, MD, USA) to enrich miRNA from 40 μg of total RNA of each sample according to manufacturer’s instructions. This kit combines a phenol/chloroform-based extraction method based on a silica membrane spin column technology. MiRNA was then reverse transcribed using the SABiosciences RT² miRNA FirstStrand Kit:331401 according to the manufacturer's protocol.

### miRNA expression profile

Array–Samples were analyzed with the SABiosciences Human miFinder RT² microRNA PCR Array, 96-well (SABiosciences Corporation) containing 352 miRNAs. One sample per group was analyzed using four different miRNA PCR array-plates, each containing different miRNAs and snord 44 as housekeeping control. Array-results were evaluated by www.sabiosciences.com/mirna_pcr_assay_search.php. The initial array simply represents a kind of decision guidance (together with the current literature) for the selection of candidates which were further investigated by real-time RT-PCR and does not have any quantitative value.

Real-time reverse transcriptase polymerase chain reaction (real-time RT-PCR)–relative quantification of specific miRNAs in tissue and blood samples was performed by real-time RT-PCR using RT² SYBR® Green qPCR Master Mix (SABiosciences Corporation) and CFX 96 real-time system Bio-Rad (Bio Rad, Munich, Germany) according to the manufacturer's protocol. Briefly, a total of 20 μl were added to each well, containing 1 μl of reverse transcribed miRNAs, 1 μl of miRNA qPCR Assay primer, 8 μl of ddH2O and 10 μl of master mix per well. Thermal protocol contained 10 min of denaturation at 95°C followed by 40 cycles of 95°C for 15 sec, 55°C for 40 sec and 72°C for 30 sec for hybridization and elongation. Real-time RT-PCR reactions were performed in triplicates. MiRNAs were considered as present when CT-values (threshold cycle) were lower than 30. Snord 44 was used as housekeeping control for normalization. Gene expression was assessed using the 2^‒ΔΔCt^ calculation method as described previously [15,16].

### Statistical Analysis

Data are presented as box plot with median (25^th^/75^th^ percentiles) and whiskers (10^th^/90^th^ percentiles). Data were found to be not normally distributed according to D'Agostino & Pearson omnibus normality test and were compared using Kruskal-Wallis test followed by Dunn´s multiple comparisons test GraphPad Prism, version 6.05, GraphPad Software, Inc., USA). A probability value of less than 0.05 was considered statistically significant.

## Results

24 Patients with carotid plaques (14 asymptomatic and 10 symptomatic patients) and 15 non-atherosclerotic internal mammary arteries were included in this study. A detailed description of the patient’s characteristics is given in Table 1. Initially, miRNA PCR array covering 352 human miRNAs was performed with in each case 3 endarterectomy samples from the carotid artery of patients with asymptomatic plaques, symptomatic plaques and samples from the unaffected internal mammary artery. Based on the microarray data as well as two previous studies showing a comparable collective of patients [17,18], we focused on miRNAs, which were easily detectable and robustly expressed across all investigated groups and have not been investigated by others before: miR-1, miR-9, miR-19b, miR-21, miR-22, miR-29b, miR-92a, miR-99a, miR-143, miR-223 and let-7f. These miRNA underwent further real-time RT-PCR analysis. In addition, we have selected the miRNAs according to the impact of their target genes on plaque growth and stability (Table 2).

**Table 2:** Target genes of the selected miRNAs and their impact on atherosclerosis.

miRNA	Target Gene (inter alia)	Source	Impact on Atherosclerosis (Source) (inter alia)
**miR-1**	KCNJ2	Yang et al., 2007, Nat Med.	Foam cell formation (Zhang et al., 2016, J Cell Mol Med.) (Zhang et al., 2016, J Cell Mol Med.)
**miR-9**	PPAR-δ	Thulin et al., 2013, Int J Mol Med.	Migration of SMCs (Marx et al., 1998, Circ Res.)
**miR-19b**	p53	Fan et al., 2014, RNA	Cell proliferation/apoptosis during plaque formation (Guevara et al., 1999, Nat Med.)
**miR-21**	MMP-9	Fan et al., 2014, Exp Mol Pathol.	Makrophage infiltration, plaque formation (Luttun et al., 2004, Circulation)
**miR-22**	Akt3	Zheng et al., 2014, Cell Physiol Biochem	Foam cell formation, plaque formation (Ding et al., 2012, Cell Metab.)
**miR-29b**	CX3CL1	Yang et al., 2015, Oral Oncol.	Macrophage infiltration, plaque formation (Teupser et al., 2004, Proc Natl Acad Sci USA)
**miR-92a**	Integrin α5	Ohyagi-Hara, 2013, Am J Pathol.	oxLDL-induced NFκB activation, monocyte adhesion (Yurdagul et al., 2014, Arterioscler Thromb Vasc Biol.)
**miR-99a**	TGF-β	Turcatel G, 2012, PLoS One	Inflammation, chemotaxis, fibrosis, proliferation, apoptosis (McCaffrey et al., 2010, Front Biosci.)
**miR-143**	COX-2	Wu et al., 2013, World J Gastroenterol.	Plaque formation (Burleigh et al., 2002, Circulation)
**miR-223**	Tissue factor	Li et al., 2014, Atherosclerosis	Plaque formation and thrombosis (Westrick et al., 2001, Circulation)
**let-7f**	TIMP-1	Egea et al., 2012, Proc Natl Acad Sci USA	MMP expression, macrophage infiltration, plaque formation (Rouis et al., 1999, Circulation)

**Fig 1:**
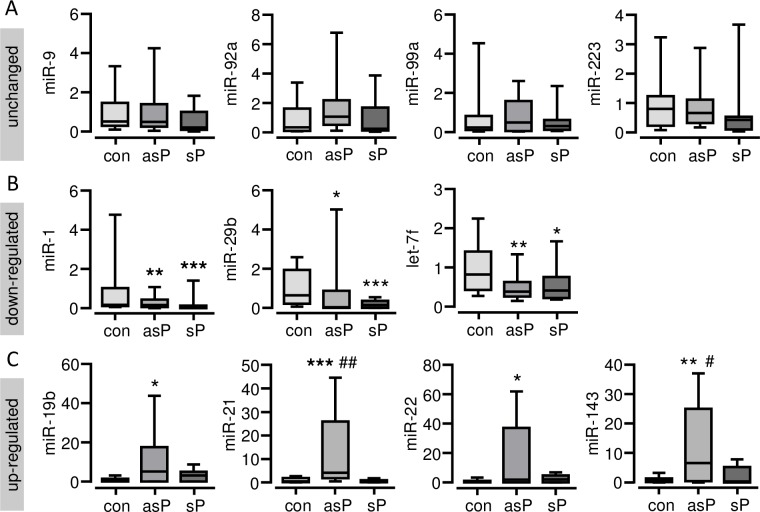
Real-time RT-PCR-analysis of miRNAs from carotid endarterectomy specimen from patients with asymptomatic and symptomatic plaques and from unaffected specimen from the internal mammary artery of healthy controls. **(A) unchanged expression, (B) down-regulated expression vs. control and (C) up-regulate expression vs. control.** Snord44 was used as housekeeping control for normalization and the relative miRNA expression was calculated using the 2^-ΔΔCT^ method. con = control, asP = endarterectomy samples of asymptomatic plaques, sP = endarterectomy samples of symptomatic plaques. Data are presented as box plot with median (25^th^/75^th^ percentiles) and whiskers (10^th^/90^th^ percentiles). *P<0.05, **P<0.01, ***P<0.001 vs. con, ^#^P<0.05, ^##^P<0.01 vs. sP

According to their expression pattern across the patient’s samples the investigated miRNAs could be classified into 3 different groups, (A) unchanged expression, (B) down-regulated expression vs. control and (C) up-regulate expression vs. control (Fig 1). Belonging to the first group no relevant differences in expression levels of miR-9, miR-92a, miR-99a and miR-223 was observed (Fig 1A). Compared to control, expression of miR-1, miR-29b and let-7f was at least 2-fold down-regulated in samples from the carotid artery of patients with asymptomatic plaques (P<0.05, P<0.01), as well as symptomatic plaques (P<0.05, P<0.001). No significant differences were seen between the patient’s groups in regard to the expression of these miRNAs (Fig 1B). The last group comprises miRNAs, which were at least 2-fold up-regulated vs. control. Interestingly, significantly up-regulated expression of miR-19b (P<0.05), miR-21 (P<0.001), miR-22 (P<0.05) and miR-143 (P<0.01) was exclusively detected in samples from patients with asymptomatic carotid plaques (Fig 1C). Whereas the expression of these miRNAs were not altered in samples from patients with symptomatic carotid plaques. Of note, expression of miR-21 (P<0.01) and miR-143 (P<0.05) was found to be significantly different between the patient’s groups (Fig 1C).

**Fig 2:**
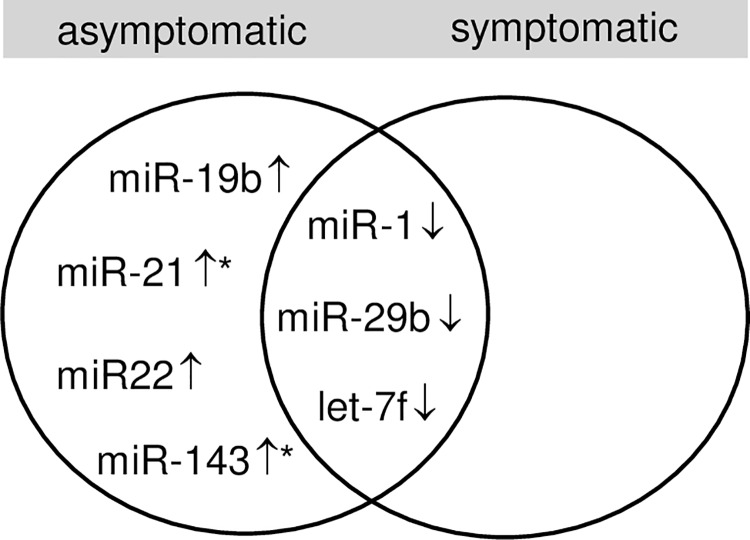
Intersection graph shows miRNAs which were significantly up- (↑) or down-regulated (↓) in endarterectomy specimen from patients with asymptomatic and symptomatic plaques vs. unaffected specimen from the internal mammary artery of healthy controls. Significant different expression between asymptomatic and symptomatic plaque samples is indicated by an asterisk (*).

Taken together, compared to control we identified miRNA, which were down-regulated in both patient’s groups and solely up-regulated solely in symptomatic samples, compared to control. Notably, miR-21 (P<0.01) and miR-143 could serve to distinguish symptomatic from asymptomatic carotid plaques (Fig 2).

## Discussion

The main focus of the present study was to analyze the expression of selected miRNAs in endarterectomy plaque specimen from patients with symptomatic and asymptomatic plaques in comparison to unaffected vascular tissue. Based on miRNA array analysis and on previous studies [17,18] we selected 11 miRNAs whose target genes thought to play an important roles in plaque growth and stability and which were further investigated by real-time RT-PCR analysis. We found several of these miRNAs differentially expressed in the collective of our patients. MiR-21 and miR-143 were identified with significant different expression levels between the groups. While they were clearly upregulated in samples from asymptomatic plaques no change was remarked in specimen from symptomatic plaques. In regard to their expression pattern, these candidates could be used to differentiate between asymptomatic and symptomatic plaques. In addition, they represent potential attractive targets to modulate plaque stability.

Over the last 10 years, miRNAs have emerged as major regulators of several pathophysiological cellular effects and signaling pathways, which crucially may contribute to the initiation and progression of atherosclerosis. These processes include lipid homeostasis, endothelial cell inflammation, leukocyte recruitment, mechanosensing and many more [19]. Even though, much progress in the understanding of miRNA function in atherosclerosis has been made during the last decade most gain of knowledge is based on experimental studies in mice. Concerning plaque stability, the widely used apolipoprotein (Apo) E or low density lipoprotein receptor (LDL-R) deficient mouse is maybe not the best object of study because in contrast to human atherosclerotic plaques, murine plaques are usually not prone to rupture [20]. Until now, only few studies exist, in which miRNA expression in human samples was investigated. In one of these few studies, Bidzhekov et al. investigated miRNA expression in blood monocyte subsets and atherosclerotic plaques in a small group of patients. They identified several miRNAs highly expressed in atherosclerotic plaques but not in healthy vessels e.g. miR-99b, miR-152 and miR-422a [21]. Furthermore, miR-145 has been described as being overexpressed in plaques from hypertensive patients [22] and plasma miR-100 has been associated with vulnerable coronary plaques in patients [23]. To the best of our knowledge, so far just two studies have been undertaken to investigate potential differences in miRNA expression between asymptomatic and symptomatic plaques in patients. Similar to our study, both studies used a rather small sample size from patients, which underwent carotid endarterectomy. They observed enhanced expression of miR-100, miR-127, miR-133a and miR-145 in symptomatic plaques [17,18]. However, control samples from unaffected arteries are lacking in these analyses. Based on our initial microarray and on these studies we selected miRNAs, which have not been investigated by other groups so far. Of note, the target genes of our candidates show a well-documented role in plaque growth and stability (Table 2).

We found none of the selected miRNAs to be up-regulated in symptomatic plaques. Interestingly, we identified miR-19b, miR-21, miR-22 and miR-143 as being up-regulated in asymptomatic plaques but not altered in symptomatic plaques. However, significant differences between asymptomatic and symptomatic plaques were only seen for miR-21 and miR-143 expression. Both miRNAs already exhibit documented contribution to atherosclerosis. MiR-21 was found to be aberrantly overexpressed in the vascular wall following experimental balloon injury in rats. Knock-down of miR-21 impeded neointima formation and decreased smooth muscle cell (SMC) proliferation [24]. Enhanced expression of miR-21 in asymptomatic plaques in our study is potentially responsible for a higher SMC content and enhanced plaque stability in those plaques. In addition, microarray analysis listed miR-21 as one of the miRNAs, which was enriched in human atherosclerotic plaques [25]. A recent study by Fan and colleagues associated miR-21 expression with coronary plaque instability and identified matrix metalloproteinase (MMP)-9 as a target gene [26]. In regard to miR-143, it has been frequently shown that the miR-143/145 gene cluster regulates the SMC phenotype, allowing SMCs to migrate and proliferate [27]. In addition, Hergenreider et al. showed that miR-143/145 is transported between endothelial cells and SMCs and was able to reduce atherosclerotic plaque burden in ApoE-KO mice [28]. Abilities of these miRNAs, in particular to regulate SMC phenotype and MMP expression could be used to increase plaque stability in patients.

In summary, we identified miR-21 and miR-143 with significant different expression levels in asymptomatic and symptomatic plaques, which may facilitate our understanding of miRNA regulation for atherosclerotic plaque stability in patients. However, we are aware of the fact that due to the limited number of ascertained miRNAs and patients included in this study the scientific significance is limited. Furthermore, due to ethical reasons, control tissues were obtained from mammary arteries, whereas atherosclerotic plaques were obtained from carotid arteries. Thus, the different environments of the selected tissues could cause potential differences in miRNA expression profile. In addition, endarterectomy samples were removed from different parts of the artery at variable positions to the stenosis, which suggests that these samples were exposed to slightly different hemodynamic flow conditions. Nevertheless, studies like ours are of particular interest to develop a specific expression pattern of miRNAs reflecting the stage and severity of atherosclerotic vascular diseases. Differential expression of regulatory miRNAs could be used as a diagnostic tool to distinguish between stable and instable atherosclerotic disease containing a high risk of plaque rupture. Advanced screening methods, such as high-density microarrays or next generation sequencing are needed for a comprehensive analysis of such patients. Validated in larger multicenter clinical trials such a disease-related specific miRNA profile could help to improve future diagnosis. Using gain-of-function (mimics) or loss-of-function (antagomirs) approaches to identify miRNAs may serve as therapeutic targets for the future treatment of patients with cerebral and coronary atherosclerotic disease.
